# Mechanism-related circulating proteins as biomarkers for clinical outcome in patients with unresectable hepatocellular carcinoma receiving sunitinib

**DOI:** 10.1186/1479-5876-9-120

**Published:** 2011-07-25

**Authors:** Charles S Harmon, Samuel E DePrimo, Eric Raymond, Ann-Lii Cheng, Eveline Boucher, Jean-Yves Douillard, Ho Y Lim, Jun S Kim, Maria José Lechuga, Silvana Lanzalone, Xun Lin, Sandrine Faivre

**Affiliations:** 1 Pfizer Oncology, La Jolla, CA, USA; 2 Beaujon University Hospital, Clichy, France; 3 Department of Internal Medicine and Oncology, National Taiwan University Hospital, Taipei, Taiwan; 4 Centre Eugène Marquis, University Hospital, Rennes, France; 5 Centre R Gauducheau, St-Herblain, France; 6 Samsung Medical Center, Seoul, Republic of Korea; 7 Korea University Guro Hospital, Seoul, Republic of Korea; 8 Pfizer Oncology, Milan, Italy; 9 Exelixis, South San Francisco, CA, USA

## Abstract

**Background:**

Several proteins that promote angiogenesis are overexpressed in hepatocellular carcinoma (HCC) and have been implicated in disease pathogenesis. Sunitinib has antiangiogenic activity and is an oral multitargeted inhibitor of vascular endothelial growth factor receptors (VEGFRs)-1, -2, and -3, platelet-derived growth factor receptors (PDGFRs)-α and -β, stem-cell factor receptor (KIT), and other tyrosine kinases. In a phase II study of sunitinib in advanced HCC, we evaluated the plasma pharmacodynamics of five proteins related to the mechanism of action of sunitinib and explored potential correlations with clinical outcome.

**Methods:**

Patients with advanced HCC received a starting dose of sunitinib 50 mg/day administered orally for 4 weeks on treatment, followed by 2 weeks off treatment. Plasma samples from 37 patients were obtained at baseline and during treatment and were analyzed for vascular endothelial growth factor (VEGF)-A, VEGF-C, soluble VEGFR-2 (sVEGFR-2), soluble VEGFR-3 (sVEGFR-3), and soluble KIT (sKIT).

**Results:**

At the end of the first sunitinib treatment cycle, plasma VEGF-A levels were significantly increased relative to baseline, while levels of plasma VEGF-C, sVEGFR-2, sVEGFR-3, and sKIT were significantly decreased. Changes from baseline in VEGF-A, sVEGFR-2, and sVEGFR-3, but not VEGF-C or sKIT, were partially or completely reversed during the first 2-week off-treatment period. High levels of VEGF-C at baseline were significantly associated with Response Evaluation Criteria in Solid Tumors (RECIST)-defined disease control, prolonged time to tumor progression (TTP), and prolonged overall survival (OS). Baseline VEGF-C levels were an independent predictor of TTP by multivariate analysis. Changes from baseline in VEGF-A and sKIT at cycle 1 day 14 or cycle 2 day 28, and change in VEGF-C at the end of the first off-treatment period, were significantly associated with both TTP and OS, while change in sVEGFR-2 at cycle 1 day 28 was an independent predictor of OS.

**Conclusions:**

Baseline plasma VEGF-C levels predicted disease control (based on RECIST) and were positively associated with both TTP and OS in this exploratory analysis, suggesting that this VEGF family member may have utility in predicting clinical outcome in patients with HCC who receive sunitinib.

**Trial registration:**

ClinicalTrials.gov: NCT00247676

## Background

Hepatocellular carcinomas (HCCs) overexpress several angiogenic proteins, including vascular endothelial growth factor-A (VEGF-A) [1-3], VEGF-D [4], and platelet-derived endothelial growth factor (PDGF) [2], as well as expressing receptors to these ligands (comprising VEGF receptors [VEGFRs]-1, -2 [5], and -3 [4]). Tumor expression of VEGF-A increases progressively during development of HCC from low-grade dysplastic nodules, and VEGF-A expression correlates with microvessel density during HCC development [6]. High serum levels of VEGF-A [7] and basic fibroblast growth factor [8] have been associated with poor clinical outcome in HCC [8], and VEGF-A polymorphisms have been associated with prognosis [9]. The hepatitis B virus X protein (HBx) is expressed in HBV-infected cells and enhances VEGF-A expression by stabilizing the transcription factor HIF-1α through inhibition of HIF-1α binding to VHL [10]. These and other findings strongly implicate angiogenesis in the pathophysiology of HCC (reviewed in [5]).

The development of sorafenib has set a precedent for the use of targeted antiangiogenic therapy in advanced HCC [11,12]. Sunitinib, an oral multitargeted tyrosine kinase inhibitor with antiangiogenic activity *in vivo*, has been investigated in advanced HCC within several phase II trials [13-15], and a phase III trial comparing sunitinib with sorafenib has recently been halted due to futility and an increased incidence of serious adverse events in the sunitinib versus the sorafenib arm. Sunitinib inhibits VEGFRs-1, -2, and -3, PDGFRs -α and -β, stem cell factor receptor (KIT), glial cell line-derived neurotrophic factor receptor (REarranged during Transfection; RET), colony-stimulating factor 1 receptor (CSF-1R), and FMS-like tyrosine kinase 3 (FLT3) [16-21]. The antiangiogenic activity of sunitinib likely results from inhibition of VEGFRs on endothelial cells and PDGFR-β on stromal cells.

Biomarkers of angiogenesis and tumor proliferation are often used to demonstrate the pharmacodynamic effects of therapeutic agents, but also have the potential to play a role in predicting which patients are likely to benefit from a particular treatment. Soluble forms of proteins involved in tumor-cell proliferation (e.g. soluble stem-cell factor receptor [sKIT]) or tumor angiogenesis (such as VEGF-A, VEGF-C, soluble VEGFR-2 [sVEGFR-2], and soluble VEGFR-3 [sVEGFR-3]) can be rapidly and readily measured in serum or plasma samples by highly specific enzyme-linked immunosorbant assays (ELISAs). If sufficiently sensitive and specific, associations between biomarker levels and clinical outcome could offer practical benefits, both for refining clinical research and for clinical decision-making.

A phase II study of sunitinib 50 mg/day on Schedule 4/2 (4 weeks on treatment, followed by 2 weeks off treatment) in 37 patients with advanced HCC was recently reported by Faivre *et al*. [14]. Although this trial did not meet its primary endpoint based on Response Evaluation Criteria in Solid Tumors (RECIST), secondary endpoints were indicative of clinical activity in this population. Median time to tumor progression (TTP) and overall survival (OS) were 5.3 and 8.0 months, respectively. Disease control rate (partial response or stable disease > 3 months) was 37.8%. In the preliminary analyses previously reported by Faivre *et al*., patients with baseline VEGF-C levels above the median achieved significantly longer TTP and OS, as well as improved disease control, compared with patients with low VEGF-C levels. This trial also investigated potential correlations between clinical outcome and other soluble proteins that are directly related to the mechanism of action of sunitinib and are associated with angiogenesis or tumor proliferation (VEGF-A, sVEGFR-2, sVEGFR-3, and sKIT). Here we report a detailed exploratory analysis of the pharmacodynamics and predictive value of these sunitinib target-related plasma proteins.

## Patients and methods

### Study design

This was a single-arm, open-label, multicenter phase II trial conducted in Europe and Asia (http://Clinicaltrials.gov identifier: NCT00247676). The study design and methods are reported in full in the primary publication of efficacy and safety data from the study [14] and summarized below.

Eligible patients were aged > 18 years with histologically proven HCC not amenable to curative surgery and a life expectancy of at least 3 months. Key inclusion criteria were: measurable disease according to RECIST [22]; Child-Pugh A or B status; Eastern Cooperative Oncology Group (ECOG) performance status of 0 or 1; and adequate liver, renal, and hematologic function. A minimum of 4 weeks was required between local therapy and disease progression for patients with recurrent or progressive disease, with resolution of all acute toxic effects of local treatment to National Cancer Institute (NCI) Common Terminology Criteria for Adverse Events (CTCAE version 3.0) grade ≤ 1 before study enrollment. Patients with previous systemic therapy for HCC were excluded. All patients provided written informed consent, and the study was conducted in accordance with International Conference on Harmonization Good Clinical Practice guidelines, the Declaration of Helsinki (1996), and applicable local regulatory requirements and laws.

Patients received a starting dose of sunitinib 50 mg/day administered orally on Schedule 4/2. Treatment continued until disease progression, unacceptable toxicity or withdrawal of consent. The primary endpoint was objective response rate; secondary objectives included evaluation of TTP, OS, and safety, and exploration of soluble plasma biomarkers. Tumor response or progression was assessed using RECIST. Changes in tumor density were evaluated in post-hoc analyses [23]. Censoring for time-to-event endpoints was based on RECIST guidelines [22].

### Assessment of biomarkers

As specified in the protocol, plasma samples for analysis of soluble proteins relevant to angiogenesis or tumor proliferation were obtained prior to the first dose on day 1, on day 14 and day 28 of cycle 1, on day 1 and day 28 of cycle 2, and on day 28 of cycle 5. The plasma samples were stored at -70°C until required for analysis. The length of storage time for the majority of samples was within the supported stability data generated during assay validation. For the samples assayed outside of their established stability, additional storage stability was evaluated at a later date to cover the duration of sample storage.

Sodium heparin plasma samples were assayed for VEGF-A, VEGF-C, sVEGFR-2, sVEGFR-3, and sKIT using validated, quantitative sandwich immunoassay ELISA kits or kit components (R&D Systems, Minneapolis, MN). sVEGFR-2, sVEGFR-3, and sKIT were each quantified with an ELISA that measured the extracellular (soluble) domain of these proteins [24]. All assays were run under Good Laboratory Practice conditions, and performance specifications of each ELISA were validated for their intended purpose. Assays were run according to the manufacturer's instructions, except in the case of sVEGFR-3, where samples were diluted 1:10 rather than 1:100 to reduce the number of samples below the limit of quantification.

### Statistical analysis

VEGF-A, VEGF-C, sVEGFR-2, sVEGFR-3, and sKIT were selected for evaluation based on their direct relevance to sunitinib's known molecular targets, on reproducible plasma pharmacodynamics in sunitinib trials in a number of tumor types, and on significant associations with clinical outcome in a particular tumor type, e.g. an association between sKIT reduction and OS in imatinib-resistant gastrointestinal stromal tumor [24-31]. With the exception of sKIT, each of these proteins has an established or putative role in VEGF-related signaling and angiogenic processes. The soluble protein analyses described here therefore represent evaluations of individual biomarker hypotheses and corrections for multiple testing were not applied.

Biomarker data were summarized using descriptive statistics. Soluble protein values that were missing at time points prior to discontinuation were excluded from the analysis. Levels of plasma proteins at baseline, and ratios to baseline levels at indicated times, were assessed for potential associations with measures of clinical outcome, including tumor response (RECIST), TTP, OS, and tumor necrosis (density reduction). For the purpose of assessing the significance of changes in plasma protein levels from those at baseline, arithmetic differences (concentration at cycle X day Y - concentration at cycle 1 day 1) were analyzed using the Wilcoxon signed-rank test. Median time-to-event (TTP and OS) values were estimated using Kaplan-Meier curves, after stratification by the median baseline plasma protein concentration or by the median plasma protein ratio to baseline at each time point. Potential correlations between soluble protein values and TTP or OS were analyzed using the Cox proportional hazards model and the log-rank test. The following applications were used for statistical analyses: Excel 2003 (Microsoft) for descriptive statistics; Prism 5.01 (GraphPad Software Inc) for the Wilcoxon signed-rank test, the Spearman rank correlation test, receiver operating characteristic (ROC) analysis, Fisher's exact test, Kaplan-Meier estimation and the log-rank (Mantel-Cox) test; and S-Plus 7.0 (Insightful) for univariate and multivariate analysis using the Cox proportional hazards model.

## Results

### Study population

Thirty-seven patients were enrolled and treated in this study. Baseline characteristics have been described in full in the per-protocol report of this trial by Faivre and colleagues [14]. The patient population was predominantly male (92%) with Child-Pugh class A liver function (84%), and all had ECOG performance status 0 or 1 (51% and 49%, respectively).

### Changes in biomarker levels during sunitinib treatment

Plasma samples were obtained from all patients on study (N = 37) at baseline and at regular time points until disease progression. For each soluble protein, there were three missing values out of 157 possible data points (1.91%), while no soluble protein values were missing at baseline. At baseline, the median (range) concentration of soluble proteins was: 54.9 (20.2-466.3) pg/mL for VEGF-A, 822.2 (334.5-3,216.5) pg/mL for VEGF-C, 7,068 (4,572.5-13,667.5) pg/mL for sVEGFR-2, 48,700 (12,420-119,300) pg/mL for sVEGFR-3 and 41,960 (17,560-85,345) pg/mL for sKIT.

**Figure 1:**
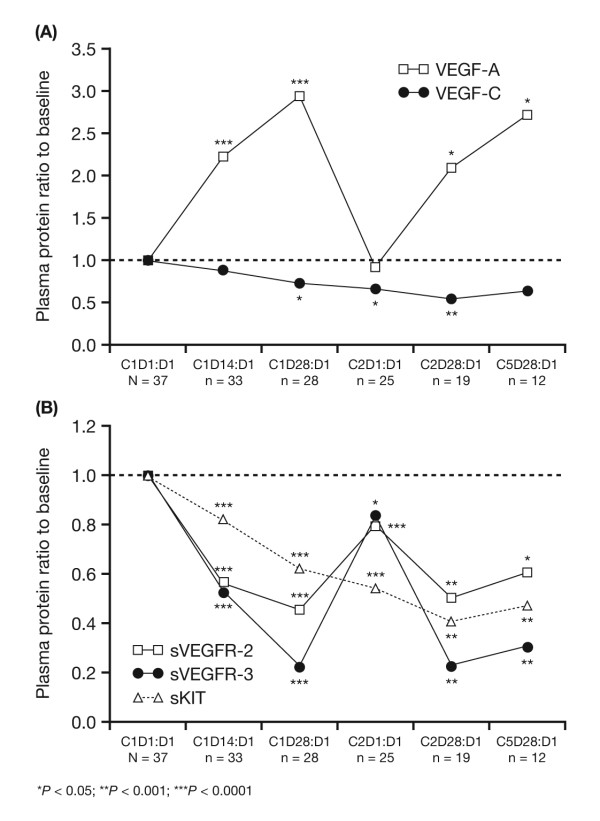
**Plasma pharmacodynamics of soluble protein biomarkers during treatment with sunitinib**. (A) VEGF-A and VEGF-C; (B) sKIT and sVEGFRs-2 and -3. C, cycle; D, day.

The median plasma level of each of the soluble proteins studied changed in response to sunitinib dosing. Significant changes from baseline in the median plasma levels of soluble proteins VEGF-A and VEGF-C and soluble receptors sVEGFR-2, sVEGFR-3, and sKIT were observed at the end of the first 4 weeks of sunitinib treatment (Figure 1). VEGF-A levels increased relative to baseline at cycle 1 day 28, while levels of all other proteins declined. The most marked changes were seen in levels of VEGF-A, which increased by 193% above baseline at cycle 1 day 28, and in sVEGFR-3, which decreased by 78.1% at the same time point. Plasma levels of sVEGFR-2 and sKIT decreased by 54.4% and 38.0%, respectively, at cycle 1 day 28. For VEGF-A, sVEGFR-2, and sVEGFR-3, these changes were partially or completely reversed during the 2-week off-treatment period, with levels returning to near baseline by the start of cycle 2. In contrast, levels of VEGF-C and sKIT declined progressively, with no return towards baseline during the off-treatment period, before leveling off at the end of cycle 2.

**Figure 2:**
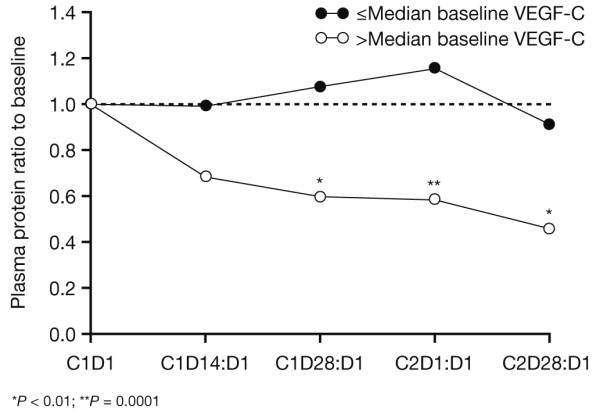
**Plasma pharmacodynamics of VEGF-C in patients with baseline VEGF-C levels above or below the median value of 822.2 pg/mL**. C, cycle; D, day.

Patients with ≤ median levels of VEGF-C at baseline had significantly lower median baseline VEGF-A (46.3 pg/mL) than patients with above-median baseline VEGF-C (94.4 pg/mL; *P *= 0.0029), and baseline concentrations of VEGF-C and VEGF-A were moderately correlated by linear regression analysis (Spearman's r = 0.6098; *P *< 0.0001). In patients with ≤ median baseline plasma VEGF-C levels, little or no change occurred in plasma VEGF-C from baseline at any time on study, whereas in patients with above-median VEGF-C at baseline, a marked reduction in VEGF-C levels was observed (Figure 2). Differences in VEGF-C ratios to baseline were significant at all time points except cycle 1 day 14. Low (≤ median) baseline VEGF-C levels were correlated with elevated VEGF-A ratios to baseline at cycle 1 day 14 (2.63 vs. 2.13, respectively; *P *= 0.0118), cycle 2 day 1 (1.27 vs. 0.86, respectively; *P *= 0.0163), and cycle 2 day 28 (5.12 vs. 1.43, respectively; *P *= 0.0014). No significant differences were seen in changes from baseline for sVEGFR-2, sVEGFR-3 or sKIT levels at any time point, after stratification by median baseline VEGF-C.

### Relationship between baseline biomarker levels and tumor response

Based on RECIST assessment of tumor response (≥ 30% reduction in unidimensional tumor size), 1 patient achieved a partial response (PR) and 13 had stable disease (SD) for > 12 weeks, yielding a disease control rate (PR or SD > 12 weeks) of 37.8% [14]. Thirteen patients (35.1%) did not experience disease control (SD < 12 weeks or progressive disease [PD]) and 10 patients were not evaluable. Analysis of tumor response using the Choi criteria (≥ 10% reduction in unidimensional tumor size or ≥ 15% reduction in tumor density) [32] was performed in 26 patients, among whom 17 patients (65.4%) were responders and 9 were non-responders according to these criteria. Table 1 and Additional File 1, Figure S1 show that patients who experienced disease control by RECIST had a significantly higher median baseline VEGF-C concentration (1,416.5 pg/mL) than those without disease control (741.5 pg/mL; *P *= 0.0027), with a trend towards higher VEGF-C levels in Choi responders vs. non-responders (*P *= 0.0662). For VEGF-A at baseline, patients with and without disease control had median baseline levels of 108.7 and 46.6 pg/mL, respectively (*P *= 0.0332) and VEGF-A levels were also significantly elevated in Choi responders (*P *= 0.0250). Baseline levels of sVEGFR-2, sVEGFR-3, and sKIT did not differ significantly when analyzed for disease control (RECIST) or by Choi response.

**Table 1:** Baseline soluble protein levels and ratios to baseline in patients stratified by clinical response (RECIST and Choi criteria)

Soluble protein and time point	RECIST					Choi criteria				
	
	Disease control		No disease control		Rank sum *P*-value	Responders		Non-responders		Rank sum *P*-value
				
	Median	n	Median	n		Median	n	Median	n	
**VEGF-A**										
Baseline, pg/mL	108.7	14	46.6	13	0.0332*	92.7	17	51.9	9	0.0250*
C2D1:D1	0.861	14	1.132	8	0.0352*	0.861	14	1.105	6	0.0757
C2D28:D1	1.426	13	3.617	6	0.0874	1.639	12	3.63	3	0.5363
**VEGF-C**										
Baseline, pg/mL	1,416.5	14	741.5	13	0.0027*	1058	17	774.8	9	0.0662
C1D28:D1	0.529	13	0.806	9	0.0708	0.595	15	1.121	7	0.0319*
C2D1:D1	0.596	14	0.947	8	0.0197*	0.5636	14	0.839	6	0.0256*
**sVEGFR-3**										
C1D14:D1	0.352	14	0.622	12	0.031*	0.4857	17	0.613	9	0.4580

Disease control (RECIST) defined as complete or partial response or stable disease > 12 weeks; no disease control defined as stable disease < 12 weeks or progressive disease

*Significant at the 0.05 level

C, cycle; D, day

**Figure 3:**
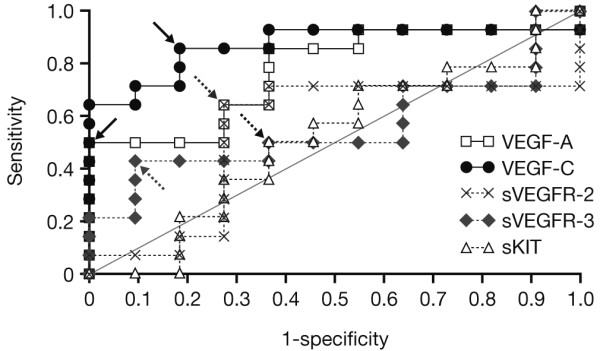
**Receiver operating characteristic (ROC) curves for prediction of disease control (partial response [PR] or stable disease [SD] > 12 weeks) by baseline level of soluble protein**. Arrows indicate ROC curve-derived cut-points.

ROC analysis was performed on baseline soluble protein levels as discriminators in predicting disease control (PR or SD > 12 weeks) versus PD, as assessed by RECIST (Figure 3). The soluble protein cut-point for response discrimination was determined from the point on the ROC curve having the minimum distance from the point corresponding to sensitivity and specificity values of 1.0. Contingency table analysis of data obtained using the ROC curve-derived cut-points revealed that baseline VEGF-C (cut-point: 942 pg/mL) was the strongest predictor of disease control, with an accuracy of 0.84 and relative risk of 4.71 (*P *= 0.0012), followed by baseline VEGF-A (cut-point: 138 pg/mL) with an accuracy of 0.72 and relative risk of 2.57 (*P *= 0.0078; Table 2). None of the soluble receptors (sVEGFR-2, sVEGFR-3 or sKIT) were significant predictors of disease control when analyzed at their ROC curve-derived cut-points.

**Table 2:** Contingency table analysis of baseline levels of biomarkers and their value in predicting disease control (complete or partial response, or stable disease > 12 weeks) vs. progressive disease with sunitinib treatment

	VEGF-A	VEGF-C	sVEGFR-2	sVEGFR-3	sKIT
Area under ROC curve, %	77.3	87.0	53.9	55.8	51.3
ROC-derived cut-point (pg/mL)	137.6	941.8	7,416	61,600	46,635
Fisher's exact *P*-value	0.0078	0.0012	0.1107	0.090	0.6887
Relative risk	2.571	4.714	1.950	1.929	1.273
Sensitivity	0.500	0.857	0.643	0.429	0.500
Specificity	1.000	0.818	0.727	0.909	0.636
Accuracy	0.720	0.840	0.680	0.640	0.560
Positive predictive value	1.000	0.857	0.750	0.857	0.636
Negative predictive value	0.611	0.818	0.615	0.556	0.500

ROC, receiver operating characteristic

### Relationship between change from baseline in biomarker levels and tumor response

Changes from baseline in levels of soluble proteins during the first two cycles of treatment were also compared between patients with and without disease control (RECIST). For VEGF-C and VEGF-A, a significant difference in change from baseline between patients with and without disease control was observed on cycle 2 day 1 (*P *< 0.05; Table 1). A reduction from baseline in median levels of each marker was seen in patients with disease control at this time point, compared with little change in those without disease control. For sVEGFR-3, the decrease from baseline was significantly greater in patients with disease control at the earliest post-baseline assessment (cycle 1 day 14; Table 1), but the difference was not significant at later time points (data not shown). Similar results were obtained when patients were stratified by Choi response criteria, although only the change in VEGF-C levels achieved statistical significance (Table 1).

### Relationship between biomarker levels and time-to-event outcomes

**Figure 4:**
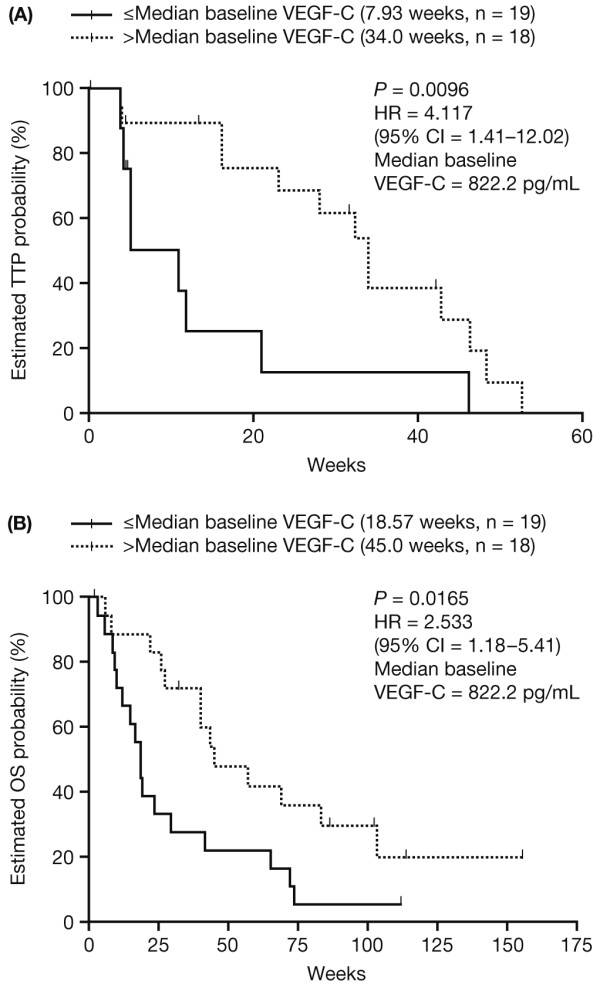
**Final Kaplan-Meier estimate of time to progression (TTP) and overall survival (OS) in patients stratified by above/below median baseline levels of VEGF-C**.

Table 3 shows median TTP and OS in patients stratified by above- or below-median plasma concentration of each biomarker at baseline. As previously reported [14], median TTP and OS were significantly longer in patients with above-median baseline levels of VEGF-C, compared with those with below-median baseline values (Kaplan-Meier curves of final TTP and OS datasets are shown in Figure 4). No other significant associations were seen between TTP or OS and baseline levels of other biomarkers.

**Table 3:** Median time to progression (TTP) and overall survival (OS) in patients stratified by above/below median baseline, and by above/below median ratio to baseline, soluble protein level.

Endpoint and soluble protein	Median baseline level, pg/mL (N = 37)	Median time to event, weeks		Log-rank *P*-value	Hazard ratio (95% CI)
				
		Patients with ≤ median baseline level (n = 19)	Patients with > median baseline level (n = 18)		
**TTP**					
VEGF-A	54.9	21.0	34.0	0.0941	2.15 (0.88, 5.25)
VEGF-C	822.2	7.93	34.00	0.0096*	4.12 (1.41, 12.02)
sVEGFR-2	7068	11.71	34.00	0.1641	1.84 (0.78, 4.33)
**OS**					
VEGF-C	822.2	18.57	45.00	0.0165*	2.53 (1.19, 5.41)
sVEGFR-3	48,700	57.00	24.64	0.0673	0.50 (0.24, 1.05)

**Endpoint, soluble protein, and time point**	**Median ratio to baseline**	**Median time to event, weeks**		**Log-rank** ***P*-value**	**Hazard ratio** **(95% CI)**
				
		**Patients with** **≤ median ratio to baseline^†^**	**Patients with** **> median ratio to baseline^†^**		

**TTP**					
***VEGF-A***					
C1D14:D1	2.2269	34.0	11.7	0.0225*	0.30 (0.11, 0.84)
C2D1:D1	0.9153	42.9	32.4	0.1341	0.44 (0.15, 1.29)
C2D28:D1	2.0923	42.9	21.0	0.0034*	0.15 (0.04, 0.53)
***VEGF-C***					
C2D1:D1	0.6596	32.43	11.71	0.0347*	0.29 (0.09, 0.92)
C5D28:D1	0.6385	48.43	34.07	0.0192*	0.16 (0.04, 0.74)
***sVEGFR-3***					
C1D28:D1	0.2195	16.14	46.29	0.0028*	5.54 (1.80, 17.02)
***sKIT***					
C1D14:D1	0.8221	34.14	16.14	0.0476*	0.33 (0.11, 0.99)
C2D28:D1	0.4067	22.00	42.86	0.1182	2.35 (0.80, 6.84)
**OS**					
***VEGF-A***					
C1D14:D1	2.2269	69.00	18.79	0.0142*	0.36 (0.16, 0.82)
C2D1:D1	0.9153	57.00	22.21	0.0862	0.45 (0.18, 1.12)
***VEGF-C***					
C1D28:D1	0.7388	45.00	21.21	0.0291*	0.37 (0.15, 0.90)
C2D1:D1	0.6596	57.00	18.57	0.0452*	0.38 (0.15, 0.98)
***sVEGFR-2***					
C1D28:D1	0.4558	20.50	71.21	0.0041*	3.96 (1.55, 10.12)
***sKIT***					
C1D14:D1	0.8221	45.00	27.50	0.1356	0.55 (0.25, 1.21)
C2D28:D1	0.4067	40.79	73.43	0.0218*	0.37 (1.21, 11.48)

Only results where *P *≤ 0.2 are shown

*Significant at the 0.05 level

^†^Number of patients included in ≤ median and > median stratification groups, respectively, at each time point: C1D14:D1: n = 17, n = 16; C1D28:D1: n = 14, n = 14; C2D1:D1: n = 13, n = 12; C2D28:D1: n = 10, n = 9; C5D28:D1: n = 6, n = 6

C, cycle; D, day

**Figure 5:**
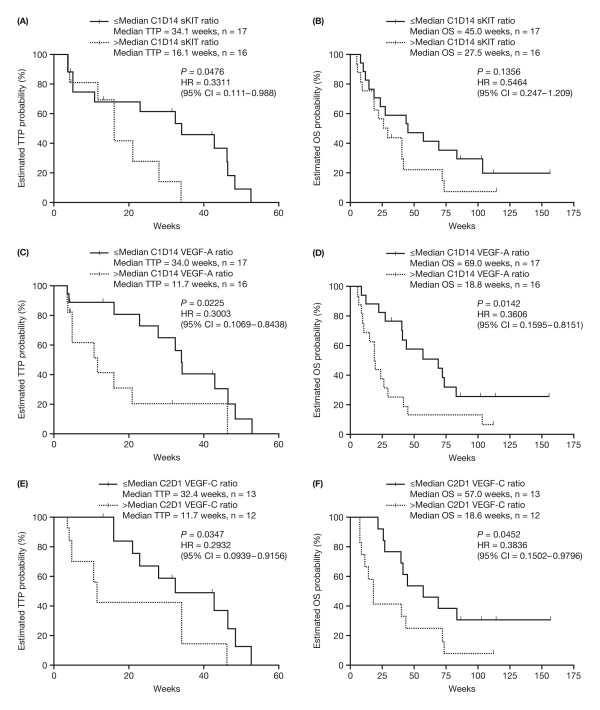
**Kaplan-Meier estimate of time to progression (TTP) and overall survival (OS) in patients stratified by above/below median ratio to baseline levels of sKIT (A and B), sVEGF-A (C and D), and VEGF-C (E and F) at post-baseline time points**. Graphs A, C, and E show TTP and graphs B, D, and F show OS. C, cycle; D, day.

Also shown in Table 3 (and Figure 5) are time-to event results for patients stratified by above- or below-median ratio to baseline at post-baseline time points. Median TTP was significantly longer in patients with ≤ median ratio to baseline of VEGF-C at cycle 2 day 1 (*P *= 0.0347) and cycle 5 day 28 (*P *= 0.0192). OS was also significantly longer in patients with ≤ median ratio to baseline of VEGF-C at cycle 1 day 28 (*P *= 0.0291) and cycle 2 day 1 (*P *= 0.0452). For VEGF-A, a similar pattern was seen, with significantly longer TTP in those with ≤ median ratio to baseline in VEGF-A at cycle 1 day 14 (*P *= 0.0225) and at cycle 2 day 28 (*P *= 0.0034), and significantly longer OS at cycle 1 day 14 (*P *= 0.0142). Above/below median ratio to baseline in soluble receptor levels each showed significant associations with TTP or OS at one or more time points (Table 3).

When soluble protein levels were analyzed as continuous variables using the Cox proportional hazards model, baseline VEGF-C was the only soluble protein significantly associated with TTP by univariate analysis (HR = 0.413; *P *= 0.0165) and showed a trend towards an association with OS (HR = 0.683; *P *= 0.190; Table 4). sVEGFR-2 ratio to baseline at cycle 1 day 28 was the only soluble protein significantly associated with OS (HR = 0.049; *P *= 0.0253). These associations remained significant for baseline VEGF-C (HR = 0.414; *P *= 0.037) and sVEGFR-2 ratio at cycle 2 day 1 (HR = 0.0257; *P *= 0.0290) by multivariate analysis of variables that were significant in univariate analyses (Table 5). In addition, ECOG performance status and Child-Pugh class were significantly associated with OS in multivariate analysis (Table 5). Notably, the proportion of patients with Child-Pugh class B disease (n = 6) was much smaller than those with class A disease (n = 31).

**Table 4:** Univariate analysis of time to progression (TTP) and overall survival (OS) using the Cox proportional hazard model

	n	TTP analysis		OS analysis	
		
		Hazard ratio (95% CI)	Log-rank *P*-value	Hazard ratio (95% CI)	Log-rank *P*-value
**Baseline characteristics**					
Age^†^	37	0.984 (0.944-1.02)	0.429	0.996 (0.962-1.03)	0.819
Sex (male vs. female)	37 (34 vs. 3)	0.214 (0.028-1.64)	0.105	0.654 (0.155-2.76)	0.559
Number of disease sites (1 vs. ≥ 2)	37 (18 vs. 19)	1.78 (0.754-4.18)	0.183	1.03 (0.501-2.11)	0.939
Cirrhosis (no vs. yes)	35 (23 vs. 12)	2.22 (0.907-5.41)	0.0743	2.23 (0.975-5.11)	0.0521
Portal vein thrombosis (no vs. yes)	37 (18 vs. 19)	1.3 (0.549-3.1)	0.547	2.00 (0.938-4.27)	0.0682
Hepatitis B (no vs. yes)	32 (15 vs. 7)	1.74 (0.685-4.4)	0.240	1.07 (0.489-2.35)	0.864
Histological grade (low or medium vs. high)	33 (22 vs. 11)	0.756 (0.276-2.07)	0.586	0.78 (0.337-1.81)	0.561
Child-Pugh class (A vs. B)	37 (31 vs. 6)	1.49 (0.428-5.18)	0.530	3.39 (1.34-8.61)	0.0065*
ECOG PS (0 vs. 1)	37 (19 vs. 18)	3.21 (1.19-8.63)	0.0157*	7.86 (2.78-22.2)	< 0.0001*
CLIP stage (≤ 2 vs. > 2)	27 (15 vs. 12)	1.57 (0.490-5.00)	0.445	1.23 (0.54-2.81)	0.62
**Soluble proteins**					
Baseline VEGF-A (ng/mL)^†^	37	0.041 (0.0006-3.00)	0.132	1.04 (0.056-19.4)	0.977
Baseline VEGF-C (ng/mL)^†^	37	0.413 (0.196-0.869)	0.0165*	0.683 (0.384-1.21)	0.190
Baseline sVEGFR-2 (ng/mL)^†^	37	0.887 (0.699-1.13)	0.325	0.969 (0.803-1.17)	0.746
Baseline sKIT (ng/mL)^†^	37	0.996 (0.959-1.04)	0.853	0.997 (0.970-1.02)	0.804
sVEGFR-2 ratio to baseline at C1D28^†^	28	0.216 (0.0084-5.54)	0.353	0.049 (0.0027-0.672)	0.0253*

Hazard ratio < 1 indicates that risk decreases with increasing value

*Significant at the 0.05 level

^†^Analyzed as continuous variables

CLIP, Cancer of the Liver Italian Program; ECOG PS, Eastern Cooperative Oncology Group performance status

**Table 5:** Multivariate analysis of variables with significant relationships with clinical outcome in univariate analysis using the Cox proportional hazard model

Variable	n	Hazard ratio (95% CI)	Log-rank *P*-value
**Time to progression**	37		
ECOG PS (0 vs. 1)		2.692 (0.987-7.34)	0.053
Baseline VEGF-C (ng/mL)^†^		0.414 (0.181-0.95)	0.037*
**Overall survival**	28		
Child-Pugh class (A vs. B)		4.053 (1.011-16.25)	0.0480*
ECOG PS (0 vs. 1)		4.875 (1.647-14.43)	0.0042*
sVEGFR-2 ratio to baseline at C1D28^†^		0.0257 (0.0001-0.681)	0.0290*

Hazard ratio < 1 indicates that risk decreases with increasing value

*Significant at the 0.05 level

^†^Analyzed as continuous variables

ECOG PS, Eastern Cooperative Oncology Group performance status

### Relationship between biomarker levels and changes in tumor density

Post-hoc analyses examined changes in tumor density on computed tomography (CT) scans during sunitinib treatment, as reported separately [23]. Twenty-six patients were assessable for changes in tumor density. For analysis of associations between protein biomarker levels and tumor density change, subjects were stratified into groups having tumor density changes at the end of cycle 1 that were above or below the median value of -31.6%, with a negative value indicating a reduction in tumor density compared with baseline (Additional File 1, Table S1). No significant associations were detected between baseline soluble protein levels and tumor density change, although there were trends towards an association between greater reductions in tumor density and high baseline levels of sVEGFR-3 or VEGF-C, and low baseline levels of sKIT. At cycle 1 day 14, greater reductions in tumor density were significantly associated with low sKIT ratios to baseline (*P *= 0.0191) and with high sVEGFR-3 ratios to baseline (*P *= 0.0221).

## Discussion

In the present study we have investigated the plasma pharmacodynamics of a number of sunitinib target-related soluble proteins and investigated potential relationships between these proteins and measures of clinical outcome, as part of a phase II study of 37 patients with advanced, unresectable HCC [14]. Potentially the most clinically useful finding from this exploratory analysis is the strong correlation between high plasma concentrations of VEGF-C at baseline and improved clinical outcome, as determined by objective response (RECIST), TTP, and OS, with baseline VEGF-C remaining an independent predictor of TTP by multivariate analysis. VEGF-C and VEGF-D are members of the VEGF family of ligands that bind to and activate VEGFR-3 [33]. Mature forms of these ligands also bind to VEGFR-2 [33], and *in vivo *angiogenic activity has been demonstrated for VEGF-C in the mouse corneal pocket assay [34]. The correlative findings for VEGF-C presented here raise the possibility that the VEGF-C/VEGFR-3 pathway may play a role in HCC disease progression, and that inhibition of this receptor may result in improved clinical outcome in a subset of patients with this disease, following treatment with sunitinib.

In support of the proposed role for the VEGFR-3 pathway in HCC progression, Thelen *et al*. [4] observed high levels of tumor cell VEGF-D expression in biopsies from HCC patients but not in specimens from cirrhotic or normal livers. VEGFR-3 was expressed in both tumor endothelium and lymphatics, suggesting that both hemangiogenesis and lymphangiogenesis may be regulated by this receptor in HCC [4]. Similar findings have been reported for VEGFR-3 expression in a number of other tumor types [35-38], and the biology of this receptor no longer appears to be restricted to lymph vessel production. When the human hepatoma cell line SKHep1, which does not express VEGF-D, was stably transfected with VEGF-D cDNA and then implanted subcutaneously in mice, larger and more metastatic tumors were formed compared with those from mock-transfected cells [4]. Interestingly, co-expression of the soluble VEGFR-3 domain in these cells blocked VEGF-D-induced tumor growth and metastatic spread.

A relationship was seen in this study between circulating VEGF-C levels prior to sunitinib dosing and the pharmacodynamics of VEGF-C and VEGF-A, but not of the soluble receptors studied. Plasma VEGF-C levels declined markedly at all time points in patients with high VEGF-C concentrations at baseline, with little change in patients with low baseline VEGF-C. This finding is consistent with the positive associations between clinical outcome and both elevated VEGF-C levels at baseline and greater reductions in VEGF-C. In contrast, sunitinib-induced increases in VEGF-A were reduced in patients with high baseline VEGF-C at some time points, suggesting an attenuated hypoxic response in this patient subset.

This is the first report in any tumor type of an association between elevated plasma levels of VEGF-C at baseline and improved clinical outcome following treatment with sunitinib. In contrast to the present finding for subjects with advanced HCC who had received no prior systemic therapy, results from a phase II study of sunitinib in patients with metastatic renal cell carcinoma (RCC) indicated that relatively low (< median) levels of VEGF-C at baseline were associated with achievement of response (RECIST) and with longer progression-free survival [39]. However, patients enrolled in this RCC study had previously progressed on bevacizumab therapy, raising the possibility that the observed biomarker correlations reflected the development of resistance to VEGF-A pathway inhibition, and no such association was seen in a phase I/II study in which patients with metastatic RCC were treated with sunitinib in combination with gefitinib [40]. It should be noted that RCC and HCC are distinct diseases that respond differently to sunitinib and that available correlative data for circulating VEGF-C in both tumors are limited, indicating a need for further research on this protein as a possible predictive biomarker in these and other tumor types.

The present exploratory analysis also showed that sunitinib dosing significantly reduced plasma sKIT from baseline levels, with no rebound during the off-treatment period. Low sKIT ratios to baseline at cycle 1 day 14 were associated with prolonged TTP and reduced tumor density, as well as with a trend towards prolonged OS. These findings support the association between sKIT reduction and improved clinical outcome reported by Zhu *et al*. in a phase II study of sunitinib in HCC [13], and suggest that inhibition of KIT signaling may contribute to sunitinib antitumor activity. The lack of early separation in the sKIT TTP and OS Kaplan-Meier curves (Figures 5A and 5B, respectively) suggests that two subsets of patients with a low sKIT ratio might exist: one that has markedly prolonged TTP and OS, and another subset with no difference. However, the relatively small sample size and higher level of censoring in the low sKIT group should be taken into consideration.

In the study by Zhu *et al*. [13], patients with HCC were treated with sunitinib at a dose of 37.5 mg/day on Schedule 4/2. The pharmacodynamics of VEGF-A, sVEGFR-2, and sVEGFR-3 were similar to those seen in the present analysis, but levels of sKIT and VEGF-C did not change significantly from baseline over 4 cycles of sunitinib treatment, in contrast to the present findings. Nonetheless, delayed tumor progression was associated with an early (day 14) decrease in circulating sKIT, consistent with the findings presented here. The possible role of KIT (CD117) in HCC is unclear. A retrospective study of archival tumor specimens from patients with histologically confirmed HCC suggested that KIT is not significantly overexpressed in this tumor type [41]. However, KIT blockade by imatinib mesylate inhibited HCC development in mice with chronic liver injury, via antiproliferative effects on KIT-expressing liver progenitor cells [42].

A number of limitations apply to the biomarker investigation reported here. Statistical analyses were not strongly powered, with plasma samples from 37 patients at baseline and declining sample sizes over time due to treatment discontinuations. Analysis of plasma proteins in relation to objective response was further limited by the proportion of patients (27.0%) not evaluable by RECIST. As this was a single-arm sunitinib study, it was not possible to determine whether biomarker associations with clinical outcome were predictive or prognostic in nature (or perhaps both). Thus, high plasma VEGF-C at baseline may represent a predictive factor for patients with HCC treated with sunitinib, consistent with potent inhibition of VEGFR-2 and -3 by this tyrosine kinase inhibitor. Alternatively, plasma VEGF-C may represent a positive prognostic factor in HCC, independent of treatment modality, as has been shown for the absence of cirrhosis in some HCC studies (reviewed in [43]). However, there are data to support high tumor VEGF-C expression as a negative prognostic factor, independent of other variables, in non-small cell lung cancer [44], esophageal cancer [45], and gastric cancer [46], while high plasma levels of VEGF-C served as an independent negative prognostic factor in colorectal cancer [47]. These findings from correlative studies in other tumor types suggest that the positive association for plasma VEGF-C in HCC reported here may be predictive rather than prognostic in nature, but further research is necessary to address this issue. The present study was limited to a small group of circulating proteins closely linked to known molecular targets of sunitinib. However, other angiogenesis-related proteins, such as basic fibroblast growth factor, as well as markers of other processes with an important role in tumor biology, such as inflammation [13], may have value in identifying patients with HCC who have inherent or acquired resistance to sunitinib therapy.

The findings reported here for selected plasma biomarkers may have value in the design of future phase III clinical trials using sunitinib in patients with HCC. In particular, a patient selection strategy that includes baseline VEGF-C concentrations above a specified value may increase the likelihood of demonstrating clinical improvement, and conversely may prevent unnecessary drug exposure in patients unlikely to benefit. Data from a phase III trial comparing sunitinib with sorafenib (NCT00699374) will soon be presented showing no advantage for sunitinib in an unselected patient population. However, identification of a subset of patients with HCC who benefit from sunitinib treatment remains an important objective of biomarker research. Furthermore, results from the present study may have relevance to the prediction of efficacy in HCC trials of drugs with a similar mechanism of action to sunitinib.

## Conclusion

In conclusion, high plasma levels of VEGF-C at baseline were strongly associated with improved clinical outcome in patients with HCC who received sunitinib, and plasma VEGF-C was an independent positive predictor of TTP by multivariate analysis. A more complete assessment of the potential clinical utility of these and other correlative findings obtained in this exploratory phase II study will require additional research.

## Competing interests

SD, ML, SL, and XL are/were all employees of Pfizer Inc. CH is an employee of Atrium Inc., owns stock in Pfizer Inc., and was a paid contractor to Pfizer Inc. in the development of this manuscript and the analysis and interpretation of data involving circulating biomarkers of angiogenesis. ER has served Pfizer Inc. in an advisory/consultancy role and J-YD has served Pfizer Inc. on an advisory board. SF has received honoraria from Pfizer Inc. All the other authors have no competing interests to declare.

## Authors' contributions

CH, SD, SL, and XL all contributed to the conception and design of the study. J-YD, HL, and JK were responsible for recruiting/supplying patients for the study trial. CH, SD, ER, ML, SL, and XL were all involved with the acquisition and interpretation/analysis of study data. A-LC was involved with the acquisition of study data. All the authors contributed to drafting and reviewing the manuscript, and all the authors read and approved the final manuscript.

## Supplementary Material

Additional file 1 **Supplementary material**. Contains Table S1 and Figure S1 (caption and artwork). Click here for file
