# Supplementary material for: Mechanism-related circulating proteins as biomarkers for clinical outcome in patients with unresectable hepatocellular carcinoma receiving sunitinib
Source: J Transl Med. 2011 Jul 25;9:120. doi: 10.1186/1479-5876-9-120 (PMC3162912; doi:10.1186/1479-5876-9-120)

**Additional File 1**

**Additional File 1, Table S1**. **Baseline levels and changes from baseline in biomarkers in patients stratified by above/below median tumor density change**

| **Plasma protein and sample time** | **Tumor density change**  **< median** | | **Tumor density change > median** | | **Wilcoxon rank sum *P*-value** |
| --- | --- | --- | --- | --- | --- |
|  | **Median biomarker level/ratio to baseline** | **n** | **Median  biomarker level/ratio to baseline** | **n** |  |
| **VEGF-A** |  |  |  |  |  |
| Baseline (pg/mL) | 93 | 13 | 54 | 13 | 0.2428 |
| C1D14:D1 ratio | 2.227 | 13 | 2.103 | 13 | 0.6866 |
| C1D28:D1 ratio | 2.708 | 12 | 3.323 | 10 | 0.3810 |
| C2D1:D1 ratio | 0.861 | 10 | 0.915 | 10 | 0.3527 |
| C2D28:D1 ratio | 1.010 | 6 | 2.092 | 9 | 0.2238 |
| C5D28:D1 ratio | 1.299 | 4 | 2.713 | 6 | 0.4762 |
| **VEGF-C** |  |  |  |  |  |
| Baseline (pg/mL) | 1,196 | 13 | 829 | 13 | 0.1534 |
| C1D14:D1 ratio | 0.951 | 13 | 0.983 | 13 | 0.4184 |
| C1D28:D1 ratio | 0.549 | 12 | 0.922 | 10 | 0.0591 |
| C2D1:D1 ratio | 0.600 | 10 | 0.622 | 10 | 0.6305 |
| C2D28:D1 ratio | 0.498 | 6 | 0.569 | 9 | 0.3884 |
| C5D28:D1 ratio | 0.478 | 4 | 0.773 | 6 | 0.6095 |
| **sVEGFR-2** |  |  |  |  |  |
| Baseline (pg/mL) | 8,542 | 13 | 7,417 | 13 | 0.4793 |
| C1D14:D1 ratio | 0.559 | 13 | 0.576 | 13 | 0.8403 |
| C1D28:D1 ratio | 0.434 | 12 | 0.510 | 10 | 0.7713 |
| C2D1:D1 ratio | 0.715 | 10 | 0.812 | 10 | 0.0753 |
| C2D28:D1 ratio | 0.513 | 6 | 0.541 | 9 | 0.6070 |
| C5D28:D1 ratio | 0.561 | 4 | 0.589 | 6 | 0.9143 |
| **sVEGFR-3** |  |  |  |  |  |
| Baseline (pg/mL) | 57,300 | 13 | 42,700 | 13 | 0.0768 |
| C1D14:D1 ratio | 0.631 | 13 | 0.304 | 13 | 0.0221* |
| C1D28:D1 ratio | 0.233 | 12 | 0.198 | 10 | 1.0000 |
| C2D1:D1 ratio | 0.801 | 10 | 0.900 | 10 | 0.7394 |
| C2D28:D1 ratio | 0.242 | 6 | 0.295 | 9 | 0.3277 |
| C5D28:D1 ratio | 0.417 | 4 | 0.279 | 6 | 0.7619 |
| **sKIT** |  |  |  |  |  |
| Baseline (pg/mL) | 39,690 | 13 | 50,665 | 13 | 0.1389 |
| C1D14:D1 ratio | 0.799 | 13 | 0.885 | 13 | 0.0191* |
| C1D28:D1 ratio | 0.615 | 12 | 0.656 | 10 | 0.3810 |
| C2D1:D1 ratio | 0.560 | 10 | 0.575 | 10 | 0.8534 |
| C2D28:D1 ratio | 0.397 | 6 | 0.502 | 9 | 0.7756 |
| C5D28:D1 ratio | 0.529 | 4 | 0.400 | 6 | 0.6095 |

*Significant at the 0.05 level

C, cycle; D, day

**Additional File 1, Figure S1.** **Scatter plot of baseline protein concentrations in patients with disease control (DC; N = 14) and without disease control (No DC; N = 13), based on Response Evaluation Criteria in Solid Tumors. Median and quartile values are indicated by bars.**


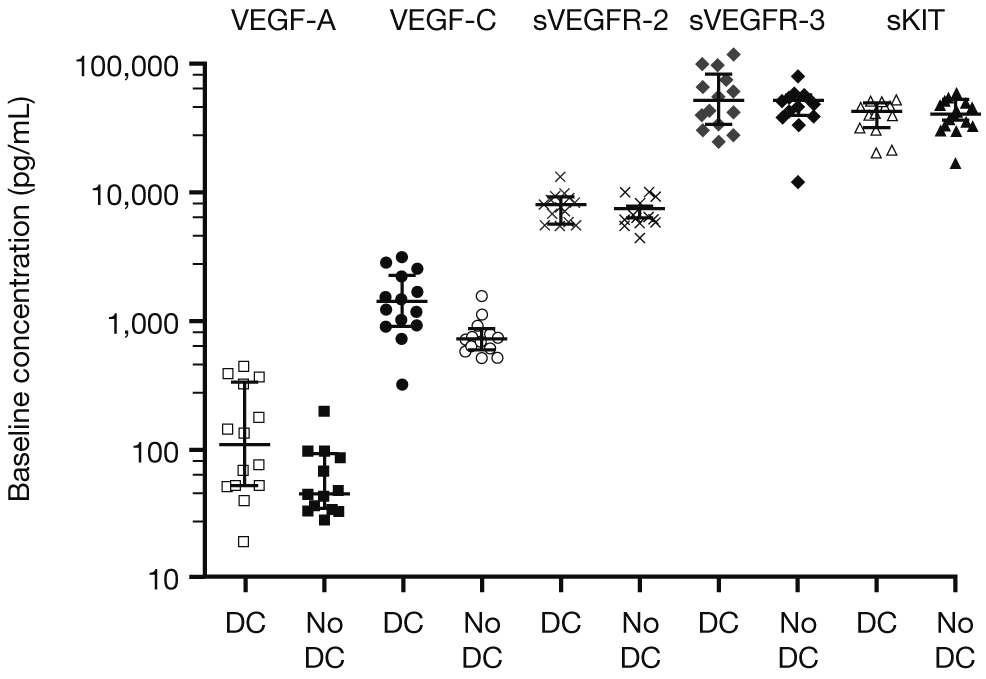

Supplement: Additional file 1 — Supplementary material. Contains Table S1 and Figure S1 (caption and artwork). [file 1479-5876-9-120-S1.DOCX]
